# The Effects of Confiding on Shift Work Nurses’ Emotion Regulation and Self-Perceived Well-Being: An Online Randomized Controlled Trial

**DOI:** 10.3390/bs15010009

**Published:** 2024-12-26

**Authors:** Cui Lu, Yawen Sun, Chunyan Wang, Tianyong Chen, Yi Tang

**Affiliations:** 1 Institute of Psychology, Chinese Academy of Sciences, Beijing 100101, China; lucui666@163.com (C.L.); sunyawen@psych.ac.cn (Y.S.); rainy.wang@126.com (C.W.); 2 Department of Psychology, University of Chinese Academy of Sciences, Beijing 100049, China; 3 Emergency Department, Tianjin Economic-Technological Development Area Hospital, Tianjin 300457, China; 4 Department of Neurology, Xuan Wu Hospital, Beijing 100053, China; tangyixw@163.com; 5 National Center for Neurological Disorders, Capital Medical University, Beijing 100053, China

**Keywords:** online intervention, confiding, nurses, nursing, well-being, emotion

## Abstract

Shift work nurses suffered great stress and emotion dysregulation during the COVID-19 pandemic. Interpersonal emotion regulation has emerged as a promising therapeutic approach, often facilitated through confiding. It has been suggested that medical staff benefit from confiding, with the act of reflecting on the social support gained from confiding being associated with higher well-being. Consequently, we hypothesized that thinking about the social support derived from confiding about work-related hassles could enhance emotion regulation and well-being in shift work nurses. This study aimed to evaluate the effects of the intervention “thinking about the social support obtained from confiding about work-related hassles” on shift work nurses’ emotion regulation and self-perceived well-being. An online randomized controlled trial was conducted with 66 shift work nurses, including 34 in the experimental group and 32 in the control group, to assess the impact of an 8-week confiding intervention focused on thinking about the social support obtained from confiding. The results indicated that the intervention significantly improved the interpersonal emotion regulation of shift work nurses in the experimental group compared to the control group. In terms of intrapersonal emotion regulation, the intervention appeared to reduce the cognitive reappraisal in the intervention group; however, there was no significant difference in cognitive reappraisal or expressive inhibition between the intervention group and control group. Furthermore, self-rated general health and sleep quality showed significant improvement in the intervention group compared to pre-test levels, but no significant differences were observed between the experimental and control groups. In conclusion, the online confiding intervention effectively enhanced interpersonal emotion regulation among shift work nurses. However, its effects on intrapersonal emotion regulation were not significant. Similarly, while participants in the intervention group reported improved self-rated general health and sleep quality, these improvements did not significantly differ from those in the control group.

## 1. Introduction

Confiding refers to the act of sharing personal information such as experiences, emotions, attitudes, and opinions with others (Slepian & Moulton-Tetlock, 2018). Many researchers, including psychological therapists, psychologists, and philosophers, have highlighted the benefits of confiding for individuals’ well-being (Slepian & Moulton-Tetlock, 2018; Pennebaker, 1997; Kelly & Mckillop, 1996; Eldridge et al., 2020; Smith, 2002; Kant, 1963; Howell et al., 2009). Studies have suggested that emotional problems, such as anxiety or depression, can be improved and alleviated through confiding (Choi et al., 2020; Liu et al., 2021). However, few studies have examined how confiding improves well-being, either from behavioral perspectives or physiological ones. From a behavioral perspective, confiding is a fundamental component of natural relationships and many psychological counseling processes, serving as a significant means for individuals to gain social support (Kelly & Mckillop, 1996; Figueiredo et al., 2004). In a before-and-after study, researchers demonstrated that thinking about the social support gained from confiding secrets significantly enhanced participants’ self-reported general well-being (Slepian & Moulton-Tetlock, 2018).

Some researchers have proposed that perceived social support serves as a mechanism of interpersonal emotion regulation to alleviate depression (Marroquín, 2011). In other words, the reason interpersonal interaction can improve mood is that it increases individuals’ perceived social support (Marroquín, 2011). Interpersonal emotion regulation refers to the regulation of one’s own emotions through interpersonal interaction (Hofmann et al., 2016; Rimé, 2007). This concept has two key features; it must occur through social interaction, and it must have a clear emotion regulation goal (Dong et al., 2024). Interpersonal emotion regulation is usually achieved through confiding (Marroquín, 2011; Hofmann et al., 2016; Rimé, 2007; Dong et al., 2024). Social support refers to the material and psychological assistance provided by social networks to individuals, helping them to reduce their stress to challenging events (Dong et al., 2024; Wu et al., 2023). It primarily includes instrumental support (material help, behavioral assistance, communication), emotional support, information support, and companionship support (Dong et al., 2024; Wu et al., 2023). Social support is inherently social and provides emotional comfort (Dong et al., 2024). It plays a crucial role in confiding behavior, particularly when confiding in friends, which yields social support benefits (Morawetz et al., 2021).

Emotion regulation is an adaptive ability, and emotion regulation strategies are learned skills (Kozubal et al., 2023) that encompass both interpersonal and intrapersonal emotion regulation strategies (Hofmann et al., 2016). Interpersonal emotion regulation refers to the regulation of one’s own emotions through the involvement of others, contrasting with intrapersonal emotion regulation (Hofmann et al., 2016; Rimé, 2007). Regarding interpersonal emotion regulation, Rimé (2007) emphasized that individuals’ emotional experiences often trigger important social behaviors such as confiding in social partners, making confiding a key approach to interpersonal emotion regulation (Rimé, 2007). In contrast, intrapersonal emotion regulation involves self-regulation strategies, which encompass five families of emotion regulation strategies: situation selection, situation modification, attention deployment, cognitive change, and response modulation (Hofmann et al., 2016; McRae & Gross, 2020; Zaki & Williams, 2013; Gross & John, 2003). Among these, cognitive reappraisal is a well-studied and widely used strategy (Hofmann et al., 2016; McRae & Gross, 2020; Zaki & Williams, 2013; Gross & John, 2003). In real-life contexts, individuals often employ mixed emotion regulation strategies (Kozubal et al., 2023; Nozaki & Mikolajczak, 2023), integrating both intrapersonal and interpersonal regulation approaches to address their emotional challenges (Zaki & Williams, 2013). The choice of strategy depends on personal habits and the specific situation at hand (Zaki & Williams, 2013). Based on this, we hypothesized that a person may rely less on intrapersonal emotion regulation if their emotional challenges have been effectively managed through interpersonal emotion regulation. While extensive research has been conducted on intrapersonal emotion regulation, there is growing interest in the interpersonal emotion regulation (Hofmann et al., 2016; McRae & Gross, 2020). Interpersonal emotion regulation represents a promising new therapeutic direction (McRae & Gross, 2020; Slepian & Moulton-Tetlock, 2018).

During the COVID-19 pandemic, various pandemic-related factors contributed to nurses’ emotion dysregulation, including shift work, burdens of overloaded and unfamiliar work, anxiety about infection, fear of transmitting the virus to their families, interpersonal isolation, and fewer interactions with friends, family, and relatives (Lim et al., 2023; Zhang et al., 2020b; Hosseini Moghaddam et al., 2022). It has been suggested that shift work diminishes emotion regulation abilities and impairs the interpersonal interactions of clinical nurses. This may be because working at night and sleeping during the day disrupts and weakens nurses’ normal social patterns. In particular, shift work nurses in China face heavier workloads and reduced team support compared to their counterparts in other contexts (Zou et al., 2021; Shen et al., 2020; Beattie et al., 2015). There was an urgent need to enhance interpersonal emotional support and other social support for shift work nurses (Morawetz et al., 2021; Kozubal et al., 2023; McRae & Gross, 2020). During the COVID-19 pandemic, shift work nurses experienced immense occupational stress, anxiety, depression, and sleep disorders (Salari et al., 2020a, 2020b; Sahebi et al., 2021; Al Maqbali et al., 2021; Marvaldi et al., 2021; Murat et al., 2021), which are indicative of typical emotion dysregulation and unsatisfied subjective well-being. Clinically, emotion dysregulation is a transdiagnostic symptom and a risk factor for psychiatric disorders (Petruso et al., 2023). It is also associated with somatic problems such as a chronic pro-inflammatory status and insomnia (Petruso et al., 2023), reflecting unsatisfied well-being. Moreover, emotion dysregulation is a key symptom of healthcare workers’ burnout. Shift work nurses’ well-being and subjective well-being at work were notably unsatisfactory, particularly during the COVID-19 pandemic (Zou et al., 2021; Beattie et al., 2015; Salari et al., 2020a, 2020b; Sahebi et al., 2021; Al Maqbali et al., 2021; Marvaldi et al., 2021; Murat et al., 2021; Petruso et al., 2023; Zhang et al., 2020a). Poor sleep quality and emotion dysregulation may be significant issues affecting the well-being and subjective well-being of Chinese shift work nurses (Zou et al., 2021; Beattie et al., 2015; Salari et al., 2020a, 2020b; Sahebi et al., 2021; Al Maqbali et al., 2021; Marvaldi et al., 2021; Murat et al., 2021; Petruso et al., 2023; Zhang et al., 2020a). This underscores the urgent need for an economical and effective method of emotion regulation for shift work nurses during the COVID-19 pandemic.

The literature on interventions aimed at reducing healthcare workers’ burnout includes individual-focused, structural or organizational, and combined interventions (Zhang et al., 2020a). Interventions that involve interpersonal emotion regulation include communication skills training, group face-to-face sessions, focus groups, and improving interaction with colleagues through personal training (Zhang et al., 2020a). However, little research has been conducted on interventions targeting confiding in natural relationships among shift work nurses, particularly on the effects of thinking about the social support gained from confiding about work-related hassles (Liu et al., 2021). Confiding in natural relationships is common and tends to have greater interpersonal influence and provide more social support than therapeutic relationships (Marroquín, 2011). Among medical staff, 79.3% reported confiding their troubles, and those who did so demonstrated lower levels of anxiety and depression (Liu et al., 2021).

The aforementioned studies and opinions suggest that reinforcing perceived social support through confiding about work-related hassles in natural relationships may serve as an effective behavioral strategy to improve shift work nurses’ emotion regulation, thereby enhancing their overall well-being. To test this hypothesis, we conducted a randomized controlled study that framed confiding about work hassles as a source of social support for shift work nurses in the intervention group. The primary aim of this study was to determine whether reflecting on social support gained from confiding could improve shift work nurses’ interpersonal emotion regulation. The primary hypothesis was that the intervention would enhance interpersonal emotion regulation while reducing reliance on intrapersonal emotion regulation, reflecting the near-transfer effects of the intervention. The secondary aim was to explore whether the intervention could improve shift work nurses’ self-rated overall well-being. The secondary hypothesis was that the intervention would improve participants’ self-rated well-being, reflecting the far-transfer effects of the intervention. Given the limitations of online interventions, we implemented an extended 8-week intervention to examine its cumulative effects over time.

## 2. Method

### 2.1. Study Design and Participants

The study was conducted online in China between December 2021 and January 2022. WeChat 7.0.8 software (WeChat, ShenZhen, China) was used to recruit participants and implement this study online. The inclusion criteria were as follows: (1) age 18–55 years, and (2) shift nurses working in public hospitals in China. The exclusion criteria were as follows: (1) individuals who could not commit to completing the experiment, (2) individuals who never or were unwilling to confide in others, (3) those considering leaving their job or planning a long holiday within the next three months, and (4) those participating in other similar psychological research. The sociodemographic characteristics of the participants are presented in the Section 3.2 (see Table 1). Participants’ consent was obtained online before the study commenced.

The sample size estimation for this intervention study was calculated based on the difference in measurements before and after the intervention. Using GPower 3.1, a total sample size of 27 would be sufficient to detect an intervention effect with α = 0.05 (one-tailed) and power = 0.80, assuming an effect size of 0.50. After completing all baseline measures, participants were randomly assigned to either the intervention group or the control group. Random allocation was conducted by the first author using Excel 2007 (Microsoft, Redmond, WA, USA). The experimental tasks were delivered one-on-one via WeChat, and the questionnaire was designed using SoJump.com (https://www.wjx.cn/ accessed on 1 March 2022) and distributed individually to participants through WeChat.

### 2.2. Intervention

#### 2.2.1. Intervention Group

Based on the theory that perceived social support serves as the mechanism of interpersonal emotion regulation to alleviate depression and that interpersonal emotion regulation is typically achieved through confiding (Marroquín, 2011; Rimé, 2007), and referencing the intervention technique developed by Slepian and Moulton-Tetlock (2018), we designed this intervention study.

Participants were instructed to maintain their usual lifestyle while recording their confiding activities over an 8-week period. Confiding was specifically defined as discussing work-related hassles for at least 5 min. During the 8-week intervention, participants were required to complete a questionnaire twice a week. The questionnaire included items on the frequency and length of confiding, the individuals they confided in, and their level of satisfaction with the confiding experience to help participants to recall their confiding activities.

Additionally, participants were provided with instructions to reflect on the social support obtained from confiding about work hassles and to record their perceived social support. Participants were asked to evaluate the amount of social support they received through confiding across three dimensions: aid, affect, and affirmation (Kahn & Antonucci, 1980). Aid consisted of three items: “problem-solving”, “receiving advice or inspiration to address issues”, and “improving the understanding of nursing care interpersonal relationships or professional knowledge”. Affect included three items: “receiving comfort or release of negative emotions”, “an increase in positive emotions”, and “enhancement of emotional connection with others”. Affirmation consisted of three items: “feeling approval or support from others”, “improving self-understanding and self-recognition”, and “enhancement of professional value recognition”. These nine items were rated on a 4-point scale, ranging from 1 (not a bit) to 4 (a lot). Participants were also asked to submit reflections on how their feelings and experiences influenced their thoughts about the social support obtained from confiding while completing the questionnaire twice a week.

#### 2.2.2. Control Group

Participants in the control group were also instructed to maintain their usual lifestyle and record their confiding about work-related hassles twice a week. Over the course of 8 weeks, these participants completed a questionnaire twice weekly. However, the questionnaire for the control group only included items about the frequency and duration of confiding, the individuals they confide in, and their level of satisfaction with the confiding experience. This approach was intended solely to help participants to recall their confiding activities.

### 2.3. Measurements

The measurements for all participants were conducted online at baseline and again after 8 weeks. The online questionnaires assessed participants’ basic information, emotion regulation, and self-rated overall health.

Participants provided data on their age, gender, marital status, number of children, education background, attitude towards confiding, confiding behavior, and financial strain (Salari et al., 2020a). Financial strain was assessed using three items from the financial strain measure: (1) “Do you have money left over almost every month?”, (2) “Do you have enough pocket money almost every month?” and (3) “Can you cover living expenses almost every month?”. If the answer to any of these questions was “no”, it was coded as 1 = having financial strain; otherwise, it was coded as 0 = no strain (Krause et al., 1991).

Emotion regulation measurements included interpersonal emotion regulation and intrapersonal emotion regulation. The Chinese version of the Interpersonal Emotion Regulation Questionnaire (IERQ) was used to measure how participants regulate their emotion through others (Hofmann et al., 2016). This version has been revised and validated for use among shift work nurses in China (Lu, 2022). The Chinese IERQ includes 14 items rated on a 5-point Likert scale and assesses three dimensions: strengthening positive emotion, seeking comfort, and social learning. Its Cronbach’s alpha value is 0.93. Higher scores indicate a higher level of interpersonal emotion regulation ability. The Chinese version of the Emotion Regulation Questionnaire (ERQ) was used to assess intrapersonal emotion regulation, covering two sub-scales: cognitive reappraisal and expressive inhibition. This questionnaire consists of 10 items rated on a 7-point Likert scale. A higher score indicated a greater use of the corresponding emotion regulation strategy (Wang et al., 2007). The Chinese version ERQ has been validated among 1163 Chinese university students, with a Cronbach’s alpha of 0.85 for cognitive reappraisal, and 0.77 for expressive inhibition.

Self-reported health included general health and sleep quality. General health was assessed using a single five-point rating scale. Sleep quality was evaluated using an item from the Pittsburgh Sleep Quality Scale (PSQI): “In general, how good do you think your sleep quality is?” (Buysse et al., 1989). Depression was detected using the Patient Health Questionnaire-9 (PHQ-9), which has a Cronbach’s alpha of 0.86 for its Chinese version (Wang et al., 2014).

### 2.4. Data Analysis

In this study, SPSS17.0 (SPSS, Chicago, IL, USA) was used for data analysis. In terms of basic information and baseline scores of study measurements, the independent samples *t*-test or chi-square test were used to examine the differences between the intervention group and the control group. When examining the difference between pre-test and post-test, a paired-samples *t*-test was used, and the effect size of the difference was calculated using Cohen’s *d*. The MANOVA was used to demonstrate the effect of the intervention by comparing the intervention group with the control group, and the results were adjusted for age and gender. The ANOVA for repeated measures was used to analyze the changes in participants’ perceived social support in the intervention group during the 8-weeks intervention period, and the results would be adjusted for age and gender.

Participants’ feelings related to thinking about the social support obtained from confiding were open-coded using the Nvivo (12.0), referred to as the primary coding. Further, the primary coding was combed, refined, and classified based on the primary coding, referred to as the secondary coding. The final subjects were extracted and purified based on the secondary coding, which is called tertiary coding.

## 3. Results

### 3.1. The Recruitment of Participants

**Figure 1:**
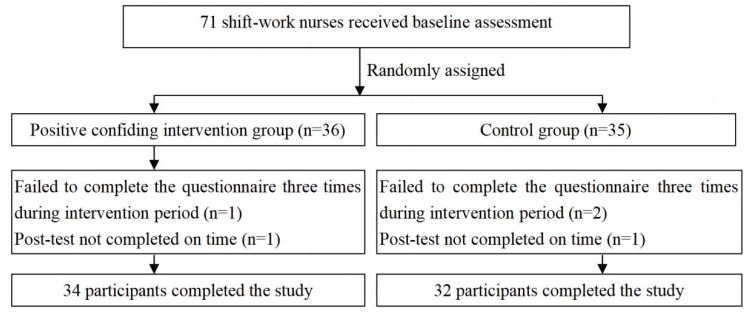
The flow chart of recruitment.

In total, 71 shift work nurses enrolled in the study. During the intervention period, two participants withdrew from the experimental group, and three withdrew from the control group. Sixty-six participants in total completed the entire study. The recruitment flow chart is demonstrated in Figure 1.

### 3.2. Basic Information

In terms of all the basic variables, there is no significant difference between the two groups (see Table 1).

### 3.3. Intervention Effects Analysis

#### 3.3.1. Social Support Gained from Confiding

**Figure 2:**
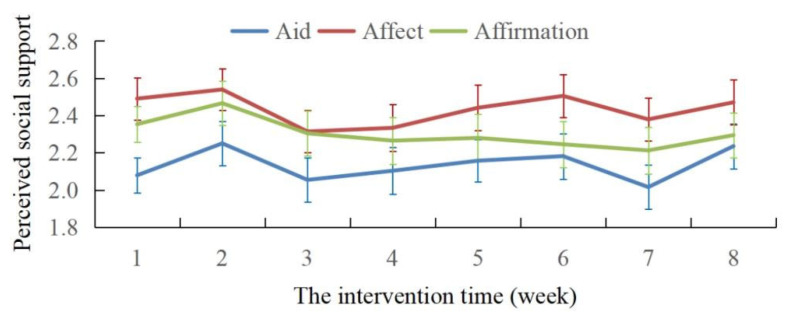
Changes in perceived social support over intervention time.

In the intervention group, the three aspects of social support include aid, affect, and affirmation (see Figure 2). The results of repeated ANOVA for 3 (aspects of social support) × 8 (weeks) showed that the interaction effect between time and social support was not significant, with *F* (1, 14) = 0.579, *p* = 0.879. The main effect of social support was not significant, *F* (1, 2) = 1.599, *p* = 0.207, while the main effect of time was significant, *F* (1, 7) = 3.056, *p* = 0.006. The results of the post hoc tests showed that there was no difference between social support in the first week and any other week, both the social support in the second week and the eighth week were higher than the third, the fourth, and the seventh week, and the social support of the sixth week was higher than the seventh week (all *p <* 0.05). Meanwhile, the score for “affect” was always the highest while the score for “aid” was always the lowest, and the results indicate that the difference between “affect” and “aid” was significant, t = 9.663, *p <* 0.001.

#### 3.3.2. Subject Analysis of Feelings of Thinking About Social Support Gaining from Confiding

Four subjects were extracted from the participants’ feelings and experiences of thinking about social support gained from confiding, including affect, affirmation, aid, and well-being benefit (see Table 2).

#### 3.3.3. Emotion Regulation

In terms of IERQ, after adjustment for age and gender, the MANOVA showed that the effect of the intervention was significant, *F* (1, 62) = 6.981, *p* = 0.010. The results of paired-samples *t*-test showed that the intervention group showed an improvement in interpersonal emotion regulation, but the difference did not reach statistical significance, *t* (33) = 1.738, *p* = 0.080, Cohen’s *d* = 0.61. The control group showed a decrease and also did not reach statistical significance, *t* (31) = 1.812, *p* = 0.092, Cohen’s *d* = 0.48 (see Table 3).

In terms of emotion regulation, MANOVA with its sub-dimensions (cognitive reappraisal and expression inhibition) was preformed respectively, and the results are shown in Table 3. In terms of cognitive reappraisal, after adjustment for age and gender, the effect of the intervention was not significant, *F* (1, 62) = 1.255, *p* = 0.267. The results of the paired-samples *t*-test indicated that the post-test cognitive reappraisal in the intervention group was significantly reduced, *t* (33) = 2.362, *p* = 0.024, with a large effect size (Cohen’s *d* = 0.82), whereas no significant changes were observed in the control group (see Table 3). For expression inhibition, after adjustment for age and gender, the effect of the intervention was not significant, *F* (1, 62) = 1.987, *p* = 0.164 (see Table 3).

#### 3.3.4. Self-Rated Well-Being Benefit

In terms of self-rated overall health, after adjustment for age and gender, the effect of the intervention was not significant *F* (1, 62) = 0.088, *p* = 0.768. The results of the paired-samples *t*-test showed that compared to the pre-test, the self-rated overall health at the post-test in both the intervention group and control group was improved (*p* < 0.05) and had a large effect size (both Cohen’s *d* > 1.0) (see Table 4).

In terms of self-rated sleep quality, after adjustment for age and gender, the effect of the intervention was not significant *F*(1, 62) = 0.520, *p* = 0.474. The results of the paired-samples *t*-test showed that compared to pre-test, the self-rated sleep quality at the post-test in both the intervention group and control group was improved at post-test (*p* < 0.05) and had a moderate effect size (Cohen’s *d* > 0.7) (see Table 4).

In terms of the results of the emotional well-being, after adjustment for age and gender, the effect of the intervention was not significant (*p* > 0.05). The results of the paired-samples *t*-test showed that compared to pre-test, there were no significant changes in the positive affect, the negative affect and the results of PHQ-9 had no significant changes (*p* > 0.05) (see Table 4).

## 4. Discussion

It has been confirmed that confiding benefits human health (Choi et al., 2020), but the conclusion depends on certain necessary conditions, including an individual’s willingness to confide, access to appropriate people to confide in, and the ability to perceive social support from confiding (Slepian & Moulton-Tetlock, 2018; Pennebaker, 1997; Kelly & Mckillop, 1996; Eldridge et al., 2020). An individual’s willingness to confide may be influenced by situational factors and personal emotion regulation strategies (Zaki & Williams, 2013). Natural relationships are the primary source of suitable confidants, though psychological counselors can also serve as effective confiding partners (Figueiredo et al., 2004). This study focused on an intervention designed to enhance participants’ perceived social support gained from confiding within natural relationships. For the first time, this intervention was applied to shift nurses to assess its effectiveness in improving interpersonal emotion regulation and self-reported well-being. This innovative approach offers a widely accessible, low-cost intervention with high ecological validity. The results demonstrated that the intervention effectively enhanced shift nurses’ interpersonal emotion regulation, although it may not have had a significant impact on their self-reported well-being.

### 4.1. Intervention Effects on Social Support

Regarding the perceived social support gained from confiding, the intervention did not result in significant changes over time across the nine items of social support. However, the qualitative analysis of the participants’ feelings about the social support gained from confiding revealed that participants reported benefits in terms of affect, affirmation, aid, and well-being. This aligns with previous studies indicating that thinking about the social support gained from confiding significantly improves participants’ self-reported well-being (Slepian & Moulton-Tetlock, 2018). This suggests that the intervention may have improved one aspect of participant’s perceived social support but not the others. Further research is needed to explore the reasons behind this phenomenon, which could provide valuable insights into mental health for shift work nurses during the COVID-19 pandemic. Additionally, within the nine items, the score for “getting comfort” was the highest, while “problem-solving” received the lowest scores. This suggests that participants received limited social support regarding problem solving, indicating a need for medical institutions to offer more social support in addressing practical work-related issues for shift work nurses. These results may also reflect the typical nature of confiding behavior, where most people initially confide for comfort, which is consistent with psychologists’ practical experience (Peck, 2020).

### 4.2. Intervention Effects on Emotion Regulation

With regard to interpersonal emotion regulation, the between-group difference was significant. The control group showed a significant decrease in interpersonal emotion regulation, while the intervention group exhibited an increase (although not statistically significant), indicating that the intervention may have improved participants’ interpersonal emotion regulation. This result supports our main research hypothesis and provided evidence for the theory that interpersonal emotion regulation enhances well-being by increasing perceived social support (Marroquín, 2011). However, due to the limited number of similar intervention studies, further research is needed to confirm this finding and compare it with these results. Moreover, the results revealed that the interpersonal emotion regulation in the control group significantly decreased, which could be attributed to the gradual onset of the COVID-19 pandemic during the intervention period. The increasing infection risk and occupational stress, coupled with decreased interpersonal interactions, may have contributed to the decline in the control group’s interpersonal emotion regulation. Additionally, because nurses often confide in order to seek social support, these findings suggest that nursing managers should listen to shift work nurses’ concerns and provide greater management and emotional support to foster organizational citizenship behaviors among shift nurses (López-Ibort et al., 2022).

Regarding intrapersonal emotion regulation, the intervention appeared to reduce participants’ cognitive reappraisal, which is inconsistent with previous studies (Lepore et al., 2000). The existing literature indicates that confiding is positively correlated with cognitive reappraisal, due to the fact that supportive responses from social network members may promote cognitive restructuring (Lepore et al., 2000). One possible explanation is that thinking about the social support obtained from confiding might reduce the mental burden on participants, leading to a lower need for cognitive reevaluation. As some participants in the intervention group noted, “I feel like I threw the garbage out! I feel lighter”. Previous studies have shown that confiding can decrease intrusive thoughts and the frequency of mind wandering over time (Lepore et al., 2000; Slepian & Moulton-Tetlock, 2018). This suggests that confiding may reduce the need for cognitive reappraisal, as cognitive reappraisal often involves mind wandering.

### 4.3. Intervention Effects on Self-Rated Well-Being

From a clinical perspective, improved interpersonal emotion regulation may benefit participants’ emotional health, which in turn could improve their self-rated overall health and sleep quality, as emotional health issues are often accompanied by somatic symptoms (Petruso et al., 2023). Accordingly, this study found that self-rated overall health and sleep quality significantly improved from post-test to pre-test, which is consistent with a previous study, although there are differences in intervention methods between the two studies (Slepian & Moulton-Tetlock, 2018). While the previous study focused on confiding secrets, our study focused on confiding work-related hassles. Both confiding secrets and confiding work-related hassles fall under the category of social sharing. The authors of the previous study argued that confiding secrets typically involves a request for help and confidentiality, compared to confiding other struggles, worries, and feelings (Slepian & Moulton-Tetlock, 2018). However, we believe that all confiding behaviors fundamentally seek social support, whether it involves seeking advice, understanding, comfort or affirmation. Additionally, although confiding work-related hassles involves personal information, it does not necessarily imply a lack of confidentiality. Therefore, we contend that confiding secrets and confiding work-related hassles are theoretically similar, and the results of the two studies are consistent and comparable.

However, this study found no between-group difference in participants’ self-rated well-being and sleep quality. One explanation for this finding is that the intervention did not affect participants’ self-rated well-being or sleep quality, and the observed intra-group differences may be attributed to the time factor. Alternatively, the intervention may have been effective in improving participants’ self-rated overall health and self-rated sleep quality, but due to study limitations, such as the sample size and the online approach, significant differences between the groups may not have been detected. Additionally, the control group was also exposed to some confiding-related questions, such as whom they confided in and their degree of satisfaction with the confiding experience. This may have prompted the control group to reflect on the social support they received and evaluate the quality of their confiding experiences, which could have led to an effect similar to that of the intervention. This might explain the lack of between-group differences, although there was an intra-group difference. Future studies address these limitations to refine the methods and improve the robustness of the findings. Meanwhile, both the between-group and inter-group differences in the results of the PHQ-9 were not significant, suggesting that the intervention did not have a substantial impact on participants’ emotional well-being compared to the control group.

## 5. Conclusions

In conclusion, this study provides a preliminary examination of the intervention “thinking about the social support obtained from confiding” among shift work nurses. The intervention used in this study was a simple, low-cost psychological regulation method, which warrants further exploration. While the results confirmed that the intervention was effective in improving shift work nurses’ interpersonal emotion regulation, its effects on intrapersonal emotion regulation, general health, and sleep quality requires further investigation. The findings and discussion in this study may have positive implications for interventions targeting mental health issues among healthcare workers.

From a theoretical perspective, the results of this study support and apply the theory of interpersonal emotion regulation, demonstrating that thinking about the social support gained from confiding enhances interpersonal emotion regulation. This aligns with Marroquín and Hofmann’s assertion that interpersonal emotion regulation improves well-being by increasing perceived social support (Hofmann, 2014; Marroquín, 2011). It is likely that social support and interpersonal emotion regulation are mutually reinforcing; however, this relationship warrants further research and discussion.

## 6. Limitation

The limitations of the present study should be acknowledged. Firstly, although the data were inspected, there may still be concerns regarding the authenticity and validity of the data collected online. Secondly, participants in the control group also reported confiding-related information, such as whom they confided in and their levels of satisfaction with the confiding process; this would have strengthened participants’ confiding behavior or prompted them to reflect on and evaluate the social support they gained from confiding, which may have had a similar effect to the intervention. This overlap in effects could explain the lack of significant differences between the two groups and represents a limitation of our study. Thirdly, due to the fact that nurses over the age of 55 rarely work night shifts in China, the participants in this study were limited to those under 55, which may restrict the generalizability of the findings to other age groups. Fourthly, the Chinese version of the ERQ used in this study was validated among Chinese university students, not shift work nurses, which is another limitation. Fifthly, emotion-regulating circuits may continue to mature until around 25 years of age, meaning that age could account for some of the variance, even though there were no significant age differences between groups. Additionally, this study was conducted between December 2021 and January 2022 in China, during a period when the government implemented policies to classify infection risks, encourage residents to stay at home, and restrict movement in medium- and high-risk areas. These measures may have decreased nurses’ interpersonal interaction, potentially influencing the results. Finally, the use of multiple t-tests in this study increased the potential for Type I error, which should be taken into consideration when interpreting the findings.

## Figures and Tables

**Table 1:** Sociodemographic characteristics of the participants.

Variables	Control Group: *n* (%)/M ± SD	Intervention Group: *n* (%)/M ± SD	*χ*^2^/*t*	*p*
1. Gender (female)	28 (87.5)	34 (100.0)	2.60	0.11
2. Age	31.09 ± 4.93	29.84 ± 5.18	1.00	0.94
3. Marital status (have a spouse)	12 (37.5)	11 (32.4)	0.27	0.65
4. Number of children			3.24	0.20
0	15 (46.9)	13 (38.2)		
1	14 (43.8)	12 (35.3)		
2 or more	3 (9.4)	9 (26.5)		
5. Education (below bachelor’s degree)	18 (43.8)	15 (55.9)	1.56	0.46
6. Having financial strain (yes)	15	20	0.95	0.46
7. Belief of the benefit to confide in others			1.09	0.58
Do not believe	10 (31.3)	9 (26.5)		
Uncertain	8 (25.0)	9 (26.5)		
Believe	14 (43.7)	16 (47.0)		
8. Frequency of confiding in others			4.36	0.36
Always (almost everyday)	0 (0)	2 (5.9)		
Often (three to four times a week)	3 (9.4)	5 (14.7)		
Sometimes (one to two times a week)	21 (65.6)	18 (52.9)		
Seldom (one to two times a month)	8 (25.0)	9 (26.5)		
9. The greatest obstacle to confiding			9.80	0.13
Confiding is not beneficial	8 (25.0)	11 (32.4)		
No one to confide to	5 (15.6)	6 (17.6)		
Worry about not being understood	4 (12.5)	4 (11.8)		
Do not want to disturb others	4 (12.5)	3 (8.8)		
Avoiding triggering negative emotions in others	4 (12.5)	5 (14.7)		
Fear of damaging relationships with others	4 (12.5)	3 (8.8)		
Other	3 (9.4)	2 (5.9)		

**Table 2:** Codes of perceived benefits from confiding.

Tertiary Coding	Secondary Coding	Primary Coding (Reference Points)
Affect	Attain comfort or release of negative emotions	Negative emotion released (53)
		Anxiety and stress reduced (27)
		Complaints reduced (1)
		Comforted (5)
	Increase of positive emotions	Mood relaxed (22)
		Positive emotions improved (43)
	Enhancement of emotional connection with others	Emotional communication enhanced (21)
		Been forgiven (1)
		Conflict reduced (1)
		Willingness to confide increased (4)
		Shared experience (10)
Affirmation	A feeling of the approval or support from others	Understanding or inspiration increased (40)
		Been heard (1)
		Been concerned about (1)
		Gained respect (1)
		Gained approval and support (21)
		Gained encouragement (5)
	Improvement of self-understanding and self-recognition	Self-understanding improved (8)
		Self-identification improved (5)
		Mutual understanding promoted (14)
	Enhancement of the professional value recognition	Operation capacity improved (21)
		Career identity improved (14)
Aid	Problem solving	Gained hands-on help (4)
		Gained more development opportunity (1)
		Helped to solve practical problems (6)
		Trouble dispelled (6)
		Mutual help promoted (2)
	Receive advice or inspiration to deal with the issues	Gained advice (4)
		Mindset adjusted (11)
		Been enlightened and persuaded (3)
		Become appreciative of life (2)
	Improving the understanding of nursing care interpersonal relationships and professional knowledge	Social comparison (9)
		Recognize the importance of communication (10)
		Transpositional consideration increased (2)
		Discussion and learning (5)
		Communication skill improved (1)
		The ability to work alongside with colleagues improved (4)
		Gained more courage to face difficulties (1)
Well-being benefit	Physical and psychological benefit	Physical and psychological health improved (1)
		Sleep improvement (3)
		Work fatigue eliminated (3)
		A lot of relaxation of body and mind (9)

**Table 3:** MANOVA on emotion regulation.

Measurements	Intervention Group (*n* = 34)				Control Group (*n* = 32)				*F*
	Pre-Test	Post-Test	*t*	Cohen’s *d*	Pre-Test	Post-Test	*t*	Cohen’s *d*	
IERQ	40.94 ± 12.92	43.26 ± 11.82	1.74	0.61	42.56 ± 12.43	38.94 ± 10.87	1.81	−0.65	6.98 *
ERQ									
Cognitive reappraisal	30.41 ± 6.28	28.06 ± 4.82	2.36 *	−0.82	27.94 ± 7.07	27.16 ± 6.58	0.83	−0.30	1.26
Expressive inhibition	16.82 ± 5.99	15.21 ± 4.05	1.74	−0.61	14.25 ± 4.67	14.81 ± 3.93	0.57	0.21	1.99

* *p* < 0.05. IERQ: Interpersonal Emotion Regulation Questionnaire (the Chinese version); ERQ: Emotion Regulation Questionnaire (the Chinese version).

**Table 4:** MANOVA on self-reported well-being.

Measurements	Intervention Group (*n* = 34)				Control Group (*n* = 32)				*F*
	Pre-Test	Post-Test	*t*	Cohen’s *d*	Pre-Test	Post-Test	*t*	Cohen’s *d*	
Self-rated overall health	2.74 (±0.56)	2.29 (±0.79)	3.28 **	1.14	2.56 (±0.91)	2.19 (±0.96)	3.48 **	1.25	0.09
Self-rated sleep quality	2.35 (±0.64)	2.12 (±0.59)	2.26 *	0.79	2.38 (±0.79)	2.06 (±0.80)	2.15 *	0.77	0.52
PHQ-9	7.44 (±4.39)	7.41 (±5.52)	0.028	0.01	6.56 (±4.10)	4.91 (±4.60)	1.35	0.48	0.94

* *p* < 0.05, ** *p* < 0.01. PANAS: The Positive and Negative Affect Scale (the Chinese version); PHQ-9: The Patient Health Questionnaire-9.

## Data Availability

The data are available upon request from the corresponding author.
